# Low lymphocyte-to-monocyte ratio and accelerated temperature rise in epidural-related maternal fever: a prospective cohort study

**DOI:** 10.1186/s12871-025-03157-0

**Published:** 2025-06-03

**Authors:** Jianxiong Huang, Yongle Li, Jiao Duan, Junjian Wen, Jingyou He, Zurong Hu

**Affiliations:** 1 Department of Anesthesiology, Guangdong Women and Children Hospital, No. 521 Xingnan Avenue, Guangzhou, Guangdong Province 511400 China; 2 Department of Anesthesiology, Guangzhou United Family Hospital, Guangzhou, Guangdong Province China

**Keywords:** Epidural-related maternal fever, Inflammatory, Lymphocyte-to-monocyte ratio, Labor epidural analgesia

## Abstract

**Background:**

Sterile inflammation is a key factor in epidural-related maternal fever (ERMF). While antepartum changes in white blood cell counts have been associated with ERMF, their impact on the occurrence of ERMF remains poorly understood.

**Objective:**

To examine the relationship between lymphocyte-to-monocyte ratio (LMR) and ERMF and to assess the potential impact of LMR on ERMF onset in parturients.

**Methods:**

This prospective cohort study included 543 parturients who underwent labor epidural analgesia at the Guangdong Women and Children Hospital from January 2022 to September 2024. ERMF was defined as a maternal temperature of ≥ 38 °C on a single occasion or two readings of ≥ 37.5 °C taken 1 h apart. Univariate and multivariate logistic regression models were utilized to explore the association between LMR level and ERMF. Receiver operating characteristic curve analysis was conducted to determine the optimal cutoff value for the LMR associated with ERMF. The Kaplan-Meier curve and log-rank test were used to compare the time to ERMF onset between parturients with higher and lower LMR levels.

**Results:**

Totally, 543parturients, 20.4% of whom developed ERMF. Lower LMR was associated with an increased incidence of ERMF (adjusted OR = 0.50, 95% CI: 0.36–0.69, *P* < 0.001). ROC curve analysis identified an antepartum maternal LMR level ≤ 2.37 as an associative cutoff for ERMF. Compared with parturients with an LMR > 2.37, those with an LMR ≤ 2.37 exhibited a significantly higher odds of developing ERMF (adjusted OR = 5.43, 95% CI: 3.18–9.28, *P* < 0.001). The onset time of ERMF was shorter in the lower LMR group but did not reach statistical significance (230.6 ± 15.3 min vs. 261.2 ± 18.4 min, *P* = 0.088). Among parturients exhibiting ERMF, a significantly higher rate of temperature rise to ERMF was observed in parturients with a lower LMR level (0.37 [0.29, 0.55] °C/h vs. 0.31 [0.20, 0.53] °C/h, *P* = 0.013).

**Conclusion:**

Low LMR levels were observed to be associated with an increased risk of ERMF.

**Trial registration:**

ChiCTR2200055734 on January 16, 2022.

**Supplementary Information:**

The online version contains supplementary material available at 10.1186/s12871-025-03157-0.

## Introduction

Labor epidural analgesia (LEA), esteemed as the benchmark for labor pain management, was first correlated with intrapartum fever in 1989 [1]. Despite initial observational studies facing criticism for potential selection bias and confounding factors, contemporary evidence has substantiated a definitive causal relationship between LEA and maternal fever [2–4]. Epidural-related maternal fever (ERMF) generally affects between 15 and 25% of patients receiving LEA during labor [5], and has been linked to various adverse outcomes [6, 7].

Notably, ERMF appears to be uniquely associated with parturients undergoing labor and has not been identified in nonlaboring women undergoing surgery with neuraxial anesthesia. This suggests that there may be an inflammatory-specific characteristic of labor related to ERMF [8]. Compared to nonlaboring parturients, laboring parturients have been shown to exhibit significantly greater increases in circulating inflammatory cytokine levels, which have been linked to an increased risk of ERMF [9]. As suggested by the sterile inflammation hypothesis, continuous infusion of local anesthetics may elevate pro-inflammatory cytokine levels at the cellular level, likely through stimulation of peripheral immune cells [10].

According to previous research, different stages of pregnancy are associated with significant changes in peripheral immune cell counts. Imbalances in immune cell ratios, such as the antepartum neutrophil-to-lymphocyte ratio (NLR) and lymphocyte-to-monocyte ratio (LMR), have been linked to the pathogenesis of various pregnancy-related disorders [11, 12]. In line with these observations, recent studies have reported that parturients who subsequently develop ERMF tend to exhibit elevated neutrophil (NEU) counts and reduced lymphocyte (LYM) counts before the onset of labor [13, 14]. The antepartum NLR has demonstrated a correlation with the incidence of ERMF [15]. However, there is currently limited knowledge on how these alterations among peripheral immune cell counts could affect the development of ERMF. Therefore, the aim of this prospective observational study was to further investigate the association of lymphocyte-to-monocyte ratio (LMR) between ERMF, and to examine how variations in the LMR influence the development of ERMF.

## Methods

### Study population

This prospective observational study was conducted at Guangdong Women and Children Hospital from January 2022 and September 2024. The study has been approved by the Ethics Committee of Guangdong Women and Children Hospital (No. 20210037) and registered at the Chinese Clinical Trial Registry (ChiCTR2200055734 on January 16, 2022). All participants provided written informed consent prior to the study. The inclusion criteria were as follows: (1) full-term singleton pregnancy (> 37 weeks); (2) planned vaginal delivery; (3) American Society of Anesthesiologists (ASA) physical status classification II or III; (4) absence of fever before labor; (5) willingness to use labor LEA and ability to provide informed consent. The exclusion criteria were: (1) parturients with specific comorbidities, including prenatal vaginitis or known group B streptococcus colonization, severe infectious diseases such as pneumonia or hepatitis, and preeclampsia; (2) parturients with contraindications to LEA; (3) body temperature ≥ 37.5 °C before LEA or administration of paracetamol within 6 h prior to receiving LEA; (4) total labor duration less than 3 h or duration of labor analgesia less than 30 min; (5) conversion to cesarean section; (6) chorioamnionitis confirmed by pathological examination; (7) incomplete case data, such as outdated complete blood count results, missing fever onset time, or undocumented timing of epidural administration.

### Implementation of LEA

LEA was initiated when contractions became regular and cervical dilation reached approximately 3 cm. Epidural puncture was performed at L3-4 or L2-3, and an epidural catheter was then inserted 2–3 cm into the epidural space. Five minutes after administering a test dose of 3 mL of 1% lidocaine via the catheter and evaluating for any side effects, an analgesic mixture of 8–10 mL of 0.1% ropivacaine with 0.5 µg/mL sufentanil was administered as a loading dose. Subsequently, the patient-controlled LEA settings were configured as follows: continuous infusion rate, 7–10 mL/h; PCA bolus, 5 mL; lockout interval, 20 min. If the visual analog scale score for pain during labor exceeded 4, an additional 5–10 mL of the same medication was administered. For parturients who did not experience adequate pain relief after this rescue dose, 5 mL of 2% lidocaine was further administered. The duration of LEA exposure was defined as the time interval from the administration of the initial loading dose of LEA to the delivery of the fetus.

### Identification of ERMF

The delivery room temperature was centrally controlled at 24–26 °C. Maternal temperature in laboring parturients was measured using a non-invasive infrared tympanic thermometer, due to its rapid readings and tolerability critical for delivery monitoring. To enhance measurement accuracy and consistency, three trained researchers performed all assessments following standardized protocols, including external auditory canal cleaning prior to the first recording. Baseline maternal temperatures were obtained before the initiation of labor epidural analgesia (LEA), and parturients with a temperature ≥ 37.5 °C were excluded from the study. Thereafter, measurements continued hourly until the delivery of the infant. ERMF was defined as a maternal temperature of ≥ 38 °C on a single occasion or two readings of ≥ 37.5 °C taken 1 h apart [16]. In cases where ERMF was diagnosed, 0.5 g of paracetamol was administered as a therapeutic intervention. The onset of ERMF is defined as the time interval from the administration of the initial loading dose of LEA to the identification of ERMF. Among parturients who developed ERMF, the temperature rise rate toward ERMF was calculated as the difference between the temperature at the identification of ERMF and the initial temperature before LEA, divided by the duration from LEA to the onset of ERMF.

### Data collection

Demographic and clinical data collected from all enrolled women included the following: maternal age, body mass index (BMI), parity of delivery, gestational age, gestational hypertension, gestational diabetes, methods of labor augmentation, prelabor rupture of membrane, duration of membrane rupture, duration of labor, body temperature before LEA, duration of LEA, ropivacaine consumption. For the purposes of this study, complete blood count results obtained within two days prior to the onset of labor were considered valid prenatal assessments. For the analysis, we selected the most recent complete blood count (CBC) results obtained within 48 h before the onset of labor. These results included WBC count, NEU count, monocyte (MONO) count, and LYM count.

### Sample size

We hypothesized that a decreased LMR is associated with an increased incidence of ERMF. Preliminary data from our pilot analysis supported this hypothesis, showing that the incidence of ERMF was approximately 40% among parturients with lower LMR, compared to about 15% in those with higher LMR. To ensure adequate statistical power for testing this hypothesis, we used PASS 15.0 software to calculate the necessary sample size. These calculations were based on an alpha level (α) of 0.05, a power (1 - β) of 0.9, and accounted for a 30% dropout rate. Given that approximately 35% of parturients were expected to have a lower LMR, our study required a minimum of 246 parturients.

### Statistical analysis

The normality of quantitative data was evaluated using the Shapiro-Wilk test, and the equality of variances was assessed with the Levene test. Normally distributed data were expressed as means and standard deviations, while non-normally distributed data were reported as medians and interquartile ranges (IQR, 25th to 75th percentiles). For comparisons between groups, the t-test was used for normally distributed data, whereas the Mann-Whitney U test was applied for non-normally distributed data as appropriate. Categorical data were presented as counts and proportions, with group comparisons performed using the chi-square test or Fisher’s exact test as appropriate. Receiver operating characteristic (ROC) curve analysis was conducted to determine the optimal cutoff value for the LMR in predicting ERMF. Covariates were initially selected using univariate logistic regression models. Multivariate logistic regression models were then employed to explore the association between LMR levels and ERMF, with results presented as odds ratios (OR) and 95% confidence intervals (CI). The Kaplan-Meier curve and log-rank test were applied to compare the time to onset of ERMF. Statistical analyses were conducted using Jamovi software version 2.6.19. Statistical significance was set at *P* < 0.05.

## Results

**Fig. 1:**
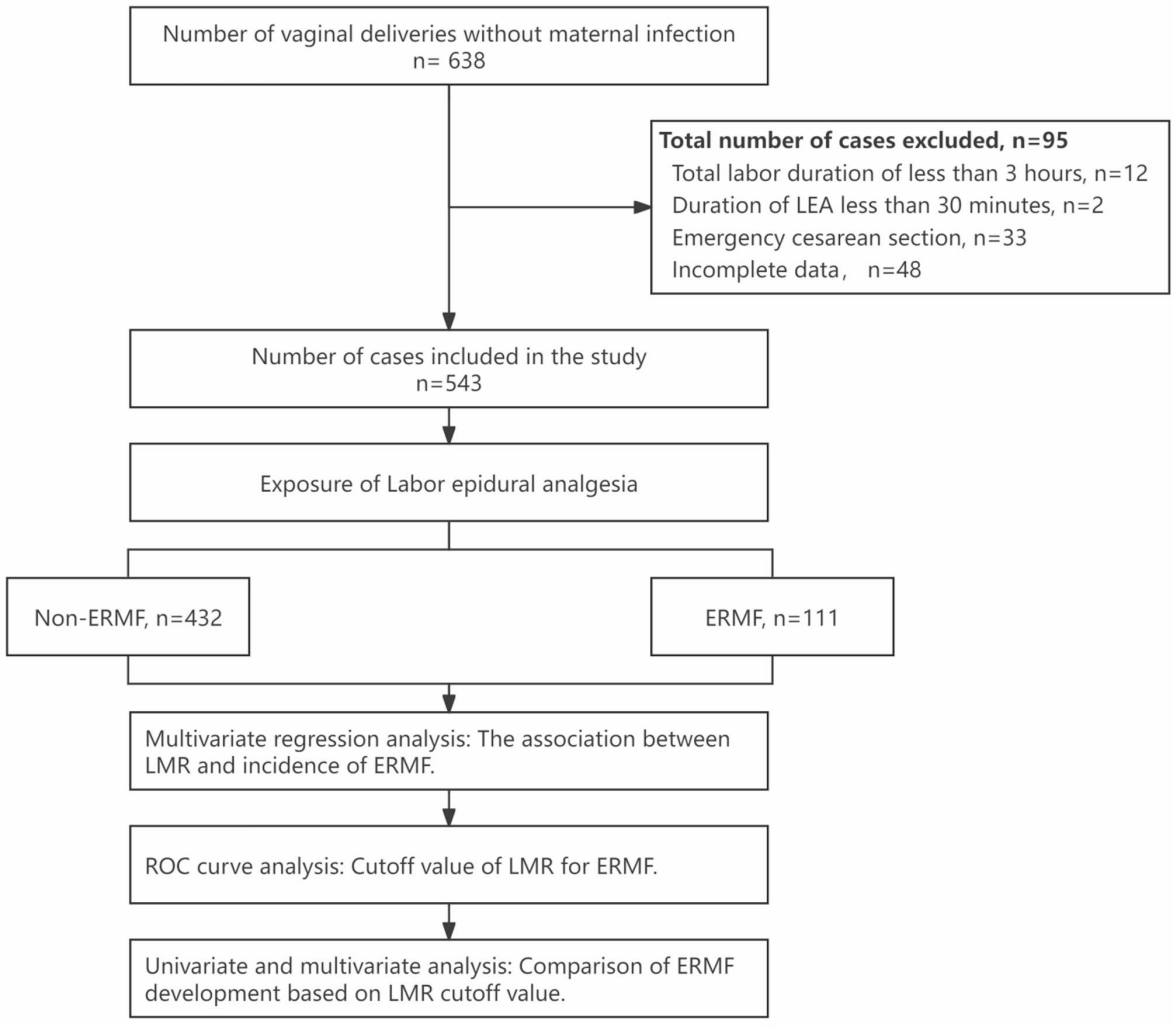
Flow Diagram for Participant Enrollment

A total of 638 parturients were initially enrolled in the study. 95 participants were subsequently excluded for the following reasons: 12 cases had a total labor duration of less than 3 h, 2 cases had a duration of LEA less than 30 min, 33 cases required an emergency cesarean section, and 48 cases had incomplete data (Fig. 1). Therefore, the final study population consisted of 543 parturients, among whom 20.4% developed ERMF.

Compared with parturients without ERMF, those who developed ERMF exhibited several notable differences (Table 1): a higher level of BMI (25.8 [25.0, 27.9] kg/m^2^ vs. 25.6 [23.7, 27.7] kg/m^2^, *U* = 20830, *P* = 0.033), a higher proportion of primiparous women (78.4% vs. 66.0%, *χ²* = 6.300, *P* = 0.012), a greater need for oxytocin (70.3% vs. 49.3%, *χ²* = 15.606, *P* < 0.001), longer duration of membrane rupture (389 [202, 740] minutes vs. 253 [105, 534] minutes, *U* = 18644, *P* < 0.001) and duration of labor (665 [520, 798] minutes vs. 456 [330, 637] minutes, *U* = 12781, *P* < 0.001), higher body temperatures before LEA (36.7 [36.6, 36.8] °C vs. 36.6 [36.5, 36.7] °C, *U* = 18447, *P* < 0.001), longer duration of LEA (388 [319, 507] minutes vs. 244 [154, 356] minutes, *U* = 11463, *P* < 0.001), and greater ropivacaine consumption (712 [548, 895] mg vs. 415 [255, 627] mg, *U* = 11466, *P* < 0.001). Additionally, parturients with ERMF demonstrated higher levels of WBC, NEU, and MONO, while showing lower levels of LYM (Table 2). After adjusting for BMI, parity, body temperature before LEA, duration of labor, oxytocin use, increased MONO counts (adjusted OR = 3.78, 95% CI: 1.01–15.03, *P* = 0.048), decreased LYM counts (adjusted OR = 0.34, 95% CI: 0.17–0.66, *P* = 0.001), and lower LMR (adjusted OR = 0.50, 95% CI: 0.36–0.69, *P* < 0.001) were each independently associated with an increased incidence of ERMF.

**Table 1:** Comparison of maternal demographic, laboratory, and intrapartum characteristics in the occurrence of ERMF

	Non-ERMF (*n* = 432)	ERMF (*n* = 111)	Statistics	*P* value
Age (years)	29 (26, 32)	29 (26, 32)	9454	0.608
BMI (kg/m^2^)	25.6 (23.7, 27.7)	25.8 (25.0, 27.9)	20,830	0.033
Gestational weeks (weeks)	39 (38, 40)	39 (39, 40)	21,404	0.069
Primipara, n(%)	285 (66.0)	87 (78.4)	6.300	0.012
Gestational hypertension, n (%)	21 (4.9)	6 (5.4)	0.055	0.814
Gestational diabetes, n (%)	70 (16.2)	12 (10.8)	2.003	0.157
Labor augmentation, n (%)				
Mechanical dilation	79 (18.3)	26 (23.4)	1.494	0.222
Oxytocin use	213 (49.3)	78 (70.3)	15.606	< 0.001
Artificial rupture of membrane	223 (51.6)	58 (52.3)	0.014	0.905
Premature rupture of membrane, n (%)	99 (22.9)	24 (21.6)	0.085	0.771
Duration of membrane rupture (min)	253 (105,534)	389 (202, 740)	18,644	< 0.001
Duration of labor (min)	456 (330, 637)	665 (520, 798)	12,781	< 0.001
Implementation of LEA				
Body temperature before LEA (°C)	36.6 (36.5,36.7)	36.7 (36.6,36.8)	18,447	< 0.001
Duration of LEA (min)	244 (154, 356)	388 (319, 507)	11,463	< 0.001
Ropivacaine consumption (mg)	415.0 (254.5, 627.0)	712.0 (547.5, 894.5)	11,466	< 0.001
Complete blood counts, 10^9^/L				
WBC	8.60 (8.03, 9.95)	9.21 (8.33, 10.4)	20,432	0.016
NEU	6.27 (5.75, 7.43)	6.94 (5.96, 8.15)	19,279	0.001
MONO	0.55 (0.50, 0.63)	0.63 (0.48, 0.72)	19,621	0.003
LYM	1.68 (1.49, 1.91)	1.43 (1.31, 1.75)	16,502	< 0.001

Abbreviations: ERMF, epidural-related maternal fever; BMI, body mass index; LEA, labor epidural analgesia; NEU, neutrophil; MONO, monocyte; LYM, lymphocyte

**Table 2:** Association of antepartum counts of NEU, MONO, LYM, and LMR levels with ERMF

	Adjusted OR (95% CI)	*P* value
NEU, 10^9^/L	1.07 (0.94, 1.21)	0.326
MONO, 10^9^/L	3.78 (1.01, 15.03)	0.048
LYM, 10^9^/L	0.34 (0.17, 0.66)	0.001
LMR, (LYM/MONO)	0.50 (0.36,0.69)	< 0.001

Abbreviations: OR, odds ratio; CI, confidence intervals; NEU, neutrophil; MONO, monocyte; LYM, lymphocyte; LMR, lymphocyte-to-monocyte ratio; ERMF, epidural-related maternal fever. ORs for each variable were separately calculated after adjusting for BMI, parity, body temperature before LEA, oxytocin use, duration of labor, duration of membrane rupture, duration of LEA, ropivacaine consumption

**Fig. 2:**
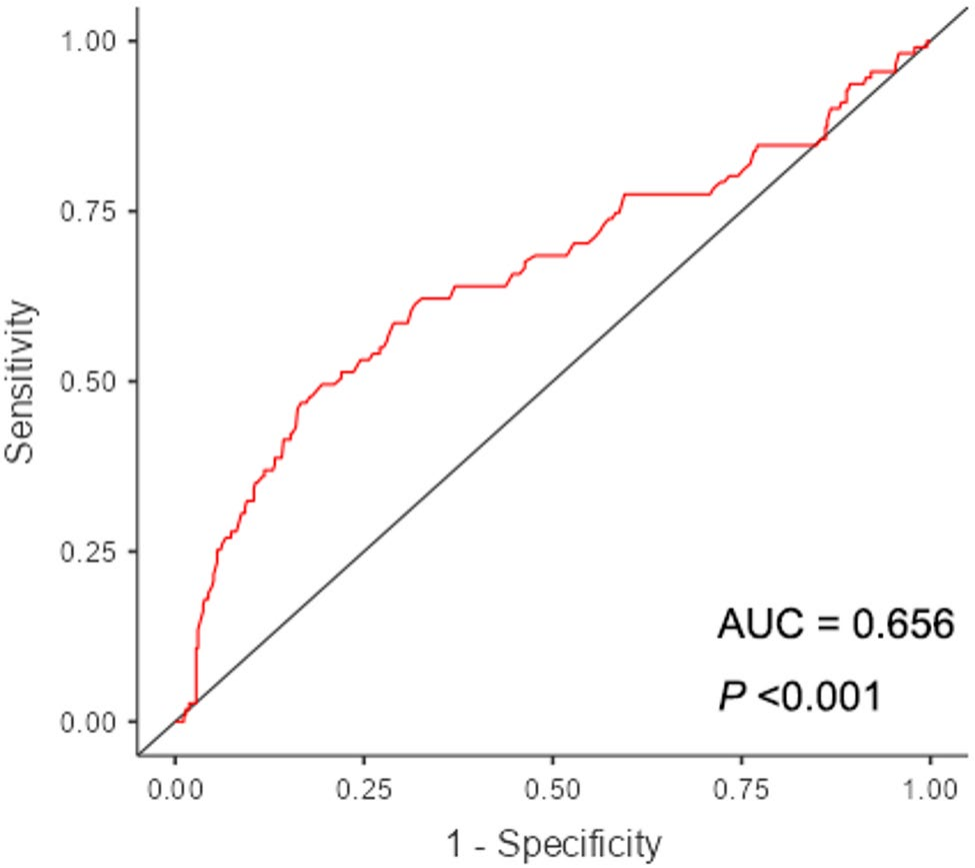
Receiver operating characteristic curve analysis for maternal LMR before epidural analgesia in predicting ERMF

ROC curve analysis identified an antepartum maternal LMR level ≤ 2.37 as an associative cutoff for ERMF (area under the curve = 0.656, Fig. 2). Compared with parturients with an LMR > 2.37, those with an LMR ≤ 2.37 exhibited a significantly higher incidence of ERMF (41.9% vs. 14,1%, *χ²* = 45.647, *P* < 0.001), corresponding to an increased risk of developing ERMF (adjusted OR = 5.43, 95% CI: 3.18–9.28, *P* < 0.001). The body temperature before LEA was comparable between the two groups, while the onset time of ERMF was shorter in the lower LMR group, but this difference did not reach statistical significance (230.6 ± 15.3 min vs. 261.2 ± 18.4 min, *χ²* = 2.948, *P* = 0.088). Among parturients exhibiting ERMF, no significant difference was found in the temperature at the detection of ERMF between those with an LMR > 2.37 and those with an LMR ≤ 2.37 (Table 3). However, a significantly higher rate of temperature rise to ERMF was observed in parturients with a lower LMR level(0.37 [0.29, 0.55] °C/h vs. 0.31 [0.20, 0.53] °C/h, *U* = 1155, *P* = 0.013).

**Table 3:** Comparison of ERMF development based on LMR cutoff values

LMR levels	Incidence of ERMF, *n* (%)	Adjusted OR (95% CI)	Temperature before LEA,°C	Temperature at ERMF detection,°C	Temperature Rise Rate,°C/h	Onset of ERMF, minutes
LMR > 2.37 (*n* = 419)	59 (14.1)	Ref	36.6 (36.6, 36.7)	38.0 (37.9,38.1)	0.31 (0.20, 0.53)	261.2 ± 18.4
LMR ≤ 2.37 (*n* = 124)	52 (41.9)	5.43 (3.18, 9.28)	36.6 (36.5, 36.7)	38.0 (37.9, 38.1)	0.37 (0.29, 0.55)	230.6 ± 15.3
Statistics	45.647	6.300	24,781	23,563	1154.5	2.948
*P*	< 0.001	< 0.001	0.422	0.107	0.013	0.088

Abbreviations: OR, odds ratio; CI, confidence interval; ERMF, epidural-related maternal fever; LEA, laobor epidural analgesia; LMR, lymphocyte-to-monocyte ratio. ORs were adjusted for BMI, parity, body temperature before LEA, duration of labor, oxytocin use, duration of labor, duration of membrane rupture, duration of LEA, ropivacaine consumption

Except for the subgroup of BMI < 25 kg/m^2^, a similar association between LMR levels and ERMF was also observed in subgroups of BMI ≥ 25 kg/m^2^ as well as other subgroup stratified by age, and parity (Table 4). Among parturients exhibiting ERMF, a significantly higher rate of temperature rise to ERMF was observed in those with a lower LMR level in the BMI ≥ 25 kg/m^2^ subgroup(0.38 [0.29, 0.55] °C/h vs. 0.31 [0.22, 0.55] °C/h, *U* = 734, *P* = 0.034), and in primipara (0.37 [0.28, 0.47] °C/h vs. 0.31 [0.20, 0.44] °C/h, *U* = 689.5, *P* = 0.030).

**Table 4:** Development of ERMF stratified by LMR levels in subgroups of age, BMI, and parity

Subgroup	Incidence of ERMF, *n* (%)	Adjusted OR (95% CI)	Temperature Rise Rate,°C/h
Age < 35 years			
LMR > 2.37 (*n* = 355)	51 (14.4)	Ref	0.31 (0.20, 0.52)
LMR ≤ 2.37 (*n* = 105)	45 (42.9)	5.63 (3.15, 10.07)	0.37 (0.28, 0.47)
Statistics	39.831	5.872	921
*P*	< 0.001	< 0.001	0.053
Age ≥ 35 years			
LMR > 2.37 (*n* = 64)	8 (12.5)	Ref	0.31 (0.18, 0.67)
LMR ≤ 2.37 (*n* = 19)	7 (36.8)	74.20 (2.97, 1854.74)	0.78 (0.48, 1.34)
Statistics	5.863	2.815	12
*P*	0.016	0.009	0.072
BMI < 25 kg/m^2^			
LMR > 2.37 (*n* = 182)	19 (10.4)	Ref	0.29 (0.19, 0.36)
LMR ≤ 2.37 (*n* = 29)	7 (24.1)	3.68 (0.99, 13.73)	0.34 (0.27, 0.38)
Statistics	4.345	1.922	44.5
*P*	0.037	0.052	0.213
BMI ≥ 25 kg/m^2^			
LMR > 2.37 (*n* = 237)	40 (16.9)	Ref	0.31 (0.22, 0.55)
LMR ≤ 2.37 (*n* = 95)	45 (47.4)	5.67 (3.06, 10.51)	0.38 (0.29, 0.56)
Statistics	33.100	5.59	734
*P*	< 0.001	< 0.001	0.034
Primipara			
LMR > 2.37 (*n* = 282)	44 (15.6)	Ref	0.31 (0.20, 0.44)
LMR ≤ 2.37 (*n* = 90)	43 (47.8)	5.89 (3.19, 10.87)	0.37 (0.28, 0.47)
Statistics	39.419	5.74	689.5
*P*	< 0.001	< 0.001	0.030
Multipara			
LMR > 2.37 (*n* = 137)	15 (10.9)	Ref	0.36 (0.24, 0.64)
LMR ≤ 2.37 (*n* = 34)	9 (26.5)	4.43 (1.47, 15.36)	0.56 (0.44, 1.28)
Statistics	5.439	2.77	40.5
*P*	0.020	0.009	0.056

Abbreviations: OR, odds ratio; CI, confidence interval; ERMF, epidural-related maternal fever; LEA, labor epidural analgesia; LMR, lymphocyte-to-monocyte ratio. ORs were adjusted for BMI, parity, body temperature before LEA, duration of labor, oxytocin use, duration of labor, duration of membrane rupture, duration of LEA, ropivacaine consumption

## Discussion

Although emerging evidence has demonstrated a correlation between changes in WBC count prior to labor, there remains a lack of research elucidating the mechanisms through which these hematological immune cell count alterations mediate the occurrence of ERMF. Our study primarily indicates that parturients with lower LMR levels are at an increased risk of developing ERMF, and further reveals that this imbalance between MONO and LYM could accelerate maternal temperature rise triggered by LEA, thereby increasing the susceptibility of parturients to ERMF.

The complex immune mechanisms underlying the initiation of labor can result in marked changes in the number and function of specific maternal immune cells, such as NEU, LYM, and MONO [17, 18, 19]. In this study, it was observed that parturients experiencing ERMF exhibited elevated MONO counts, and reduced LYM counts. The LMR serves as a sensitive composite marker of inflammation, with a reduction indicating an imbalance between MONO and LYM counts [20, 21, 22]. Substantial evidence from previous studies shows that parturients experiencing ERMF often exhibit decreased LYM counts [13, 14, 15]. Given the critical role of helper T lymphocytes in modulating immune responses [23, 24], a reduction in maternal lymphocyte levels may hinder the release of anti-inflammatory factors such as interleukin-10 (IL-10) [25, 26]. Conversely, elevated MONO counts contribute to increased production of pro-inflammatory cytokines like IL-1β, IL-6, IL-8, and TNF-α, thereby enhancing the overall inflammatory response [27, 28, 29]. Therefore, parturients with a reduced prepartum LMR are likely to have elevated levels of pro-inflammatory cytokines such as IL-6 and IL-8, alongside decreased levels of the anti-inflammatory cytokine IL-10. These characteristics align with the maternal inflammatory profiles observed in parturients who subsequently develop ERMF [30, 31, 32].

Based on these findings, we proceeded to perform a multivariate regression analysis with LMR ≤ 2.37 designated as the exposure variable. The findings indicated that lower antepartum LMR is associated with an increased risk of ERMF. Considering the findings from an adequately powered randomized controlled trial showing that prophylactic broad-spectrum antibiotics did not prevent the onset of ERMF [33], sterile inflammation is now recognized as a key factor in ERMF development. Commonly used local anesthetics in epidural analgesia, such as ropivacaine and bupivacaine, have been shown to influence inflammatory responses: ropivacaine can induce cellular apoptosis and increase the secretion of IL-6 and IL-8 in a dose-dependent manner [34, 35], while bupivacaine amplifies the pro-inflammatory effect of IL-1β by reducing caspase-1 activation and increasing intracellular monocyte counts, thereby mediating fever [36]. Given that the inflammatory response triggered by local anesthetics requires time to synthesize and release cytokines, most studies indicate that ERMF typically manifests within 6 h of initiating LEA [37, 38, 39]. In this study, it was observed that parturients with a lower antepartum LMR exhibited a notably faster rate of temperature increase and tended to show an earlier onset of ERMF. However, this trend did not reach statistical significance. The absence of statistically significant differences in the time to ERMF onset may be attributable to the relatively small sample size utilized in this investigation. The accelerated temperature rise observed in parturients with a lower LMR may have clinical implications. Previous studies have reported that ERMF typically manifests 6 h after the initiation of epidural analgesia. However, in individuals with an elevated temperature increase rate, the threshold for fever could be reached earlier, potentially shortening the window for timely intervention. This subgroup of patients may benefit from more frequent temperature monitoring to mitigate risks to both maternal and fetal outcomes.

Consistent with previous studies, our study reported an incidence of ERMF of 20.4%, while a significantly higher incidence of 41.9% observed in parturients with LMR ≤ 2.37. Moreover, the incidence of ERMF in parturients with LMR > 2.37 appeared comparable across different subgroups. However, the incidence of ERMF in parturients with LMR ≤ 2.37 was observed to be higher in the subgroups of age < 35 years, age ≥ 35 years, BMI ≥ 25 kg/m², and primipara, compared to the subgroups of BMI < 25 kg/m² and multipara. Furthermore, the effect of an accelerated rise in temperature associated with lower LMR was more pronounced in the subgroups of BMI ≥ 25 kg/m² and primipara. These findings suggest that individual characteristics may influence the association between LMR and ERMF. Notably, although LMR showed a statistically significant association with ERMF in this study, its discriminatory ability was only moderate, with an AUC of 0.656. Therefore, this cutoff value should be interpreted with caution and not used in isolation for clinical decision-making. Integrating LMR with other clinical indicators may improve risk prediction, and future studies are needed to explore more robust biomarkers or composite models for better stratification of parturients at risk for ERMF.

Distinct from prior research, our study aims to investigate the relationship between prepartum alterations in the LMR and the development of ERMF, with a specific focus on the rate of maternal temperature rise. Several limitations of this study warrant consideration. First, as an observational cohort study conducted at a single center, our findings are inherently subject to potential confounding and limited generalizability. Second, The use of tympanic thermometry, while non-invasive, may underestimate low-grade or transient fevers clinically relevant to ERMF. Additionally, hourly temperature recording instead of continuous monitoring could further limit the detection of early fever onset, potentially affecting the accuracy of ERMF incidence and timing estimates. Third, we did not routinely assess changes in the sensory block level during labor epidural analgesia; therefore, fluctuations in the anesthesia plane could not be excluded as a contributing factor to ERMF. Despite these limitations, our study highlights the potential value of LMR as a predictive marker for ERMF risk and contributes to the ongoing efforts to identify modifiable factors associated with this condition. Future prospective, multicenter studies with larger sample sizes and more refined monitoring protocols are needed to confirm these findings and further explore the underlying mechanisms.

## Conclusion

Low LMR levels were observed to be associated with an increased risk of ERMF and a faster maternal temperature rise following labor epidural analgesia in this observational study. These findings suggest a potential role of LMR in maternal inflammatory responses related to ERMF. However, further research is needed to confirm causality and explore underlying mechanisms.

## Electronic supplementary material

Below is the link to the electronic supplementary material.

Supplementary Material 1

Supplementary Material 2

Supplementary Material 3

## Data Availability

All data generated or analysed during this study are included in the supplementary information files.

## Abbreviations

LEA: Labor epidural analgesia
ERMF: Epidural-related maternal fever
WBC: White blood cell
NEU: Neutrophil
MONO: Monocyte
LYM: Lymphocyte
LMR: Lymphocyte-to-monocyte ratio
BMI: Body mass index
ROC: Receiver operating characteristic
OR: Odds ratio
CI: Confidence interval
