# Supplementary material for: Low lymphocyte-to-monocyte ratio and accelerated temperature rise in epidural-related maternal fever: a prospective cohort study
Source: BMC Anesthesiol. 2025 Jun 3;25:283. doi: 10.1186/s12871-025-03157-0 (PMC12131660; doi:10.1186/s12871-025-03157-0)
Supplement: Supplementary file 1 — Supplementary Material 1 [file 12871_2025_3157_MOESM1_ESM.pdf]

Guangdong Women and Children Hospital Ethical Clinical Research Project Establishment Review Resolution

Project Name	Analysis of Risk Factors for Fever Related to Epidural Analgesia During Labor and the Predictive Value of LMR				
Number	Guangdong Women and Children Hospital Clinical Research [20210037]				
Investigator	Yongle Li	Department	Anesthesiology	Project Category	Clinical Observational Study

Ethics Committee Review Resolution:

The Ethics Committee has reviewed the project titled "Analysis of Risk Factors for Fever Related to Epidural Analgesia During Labor and the Predictive Value of LMR" submitted by Dr. Li Yongle from the Department of Anesthesiology. The committee finds that the research content and methods adhere to the ethical standards and requirements for clinical research and hereby approves the initiation of this project at our hospital.

东省妇幼保健院 学术委员会

主任委员签名 [illegible]

2021年 11月 26日
